# Influence of contrast compression therapy and water immersion contrast therapy on biomechanical parameters of the forearm muscles in martial arts athletes

**DOI:** 10.3389/fphys.2025.1494762

**Published:** 2025-04-10

**Authors:** Robert Trybulski, Jarosław Muracki, Robert Roczniok, Wacław Kuczmik, Nicola Lovecchio, Adrian Kużdżał

**Affiliations:** ^1^ Medical Department, Wojciech Korfanty Upper Silesian Academy, Katowice, Poland; ^2^ Provita Medical Center, Żory, Poland; ^3^ Institute of Physical Culture Sciences, Department of Physical Culture and Health, University of Szczecin, Szczecin, Poland; ^4^ Institute of Sport Science, Jerzy Kukuczka Academy of Physical Education in Katowice, Katowice, Poland; ^5^ Department of General Surgery, Vascular Surgery, Angiology and Phlebology, Medical University of Silesia, Katowice, Poland; ^6^ Department of Human and Social Sciences, University of Bergamo, Bergamo, Italy; ^7^ Institute of Health Sciences, College of Medical Sciences, University of Rzeszów, Rzeszów, Poland

**Keywords:** regeneration, readiness to play, MMA fighters, water immersion, contrast therapy, myotonometry, pressure pain threshold

## Abstract

**Objective:**

This study compared the immediate effects of game-ready contrast therapy (GRT) and contrast water immersion therapy (CWT) on stiffness, muscle tone, flexibility, pressure pain threshold, and isometric muscle strength.

**Design:**

Experimental, single-blind, randomized controlled trial. Thirty volunteers training MMA (age: 28.20 ± 7.57 years, BMI: 26.35 ± 4.06, training experience: 10.37 ± 7.34) were randomized to two groups: experimental (n = 15) and control (n = 15). In the first phase, the experimental group underwent GRT and the control–game-ready sham therapy (GRS). After a 2-week break, the experimental group underwent CWT and the control–contrast water sham therapy (CWS). The main outcome measures were muscle tone (T) stiffness (S) elasticity (E), pressure pain threshold (PPT), and maximum isometric strength (Fmax) assessed before therapy (Rest) and 5-min and 1-h after treatment (PostTh5min and PostTh1h).

**Results:**

Analysis of variance results for T, S, E, PPT, and Fmax showed statistically significant differences (p < 0.0001) for main effects and interactions. For both therapies GRT and CWT: T, S, and E were lower 5 min after therapy and 1 h after therapy compared to Rest (interaction effect, p < 0.00001). For both therapies GRT and CWT the PPT and Fmax were higher 5min and 1 h after therapy compared to Rest (interaction effect, p < 0.0001). The *post hoc* test showed statistically significant differences (p < 0.0001) for T, S, E, PPT, and Fmax in the experimental groups (GRT and CWT) for Rest-PostTh5min and Rest-Post1h. No statistically significant differences were found for Post5mi-Post1h. The effect size of Cohen’s d for S, E, PPT, and Fmax showed similar values, with only T being significantly more pronounced in the GRT group (large, d > 0.8). There were no statistically significant differences (p > 0.05) in the control groups (GRT for GRS and CWT for CWS) in the Rest-PostTh5min-PostTh1h range.

**Conclusion:**

The positive impact of both contrast therapy strategies as a stimulus influencing important aspects of biomechanics was confirmed. The results showed similar effects of CWT and GRT (both similarly lowering S and E and increasing Fmax and PPT) except for the analysis of muscle tone, where the lowering effect of GRT had larger effect. These findings can be directly applied by researchers, sports medicine specialists, and martial arts trainers interested in the biomechanical effects of therapy on athletes, improving their understanding and practice.

## Introduction

A common practice among athletes is using various regeneration methods, the main goal of which is to offset the adverse effects of excessive physical exercise (Enoka, 1997). Excessive load on skeletal muscles can cause mechanical damage to cell membranes (Kelly et al., 2018), activation of inflammatory processes (Jones et al., 1986), increased muscle soreness (Dupuy et al., 2018), as well as increased muscle stiffness, elasticity, and tone (Trybulski et al., 2024a). Changes in the biomechanical parameters of the muscles impact the neuromuscular processes and impair motor control–some of the damaged muscle fibers cannot generate the optimal twitch, and the firing of the motor units changes. Consequently, damage to the fibers and changes in the biomechanical properties of muscles may affect sports performance (Zebrowska et al., 2019) and cause injury to the athlete (James, 2014). Some sports disciplines excessively use forearm muscles (Alm and Yu, 2013). In this group, we consider combat sports (mixed martial arts [MMA], Brazilian jujitsu [BJJ], judo [J]), in which the grip strength generated by the forearm muscles plays a crucial role (Zebrowska et al., 2019). Indeed, the repetitive concentric, eccentric, and isometric contractions of these muscle groups utilized during chokes, throws, holds, and punches require special training and appropriate regeneration (Andrade et al., 2019).

In point of this, contrast therapy becomes one of the regenerative methods used in clinical practice (Nahon et al., 2021). One method of contrast therapy is immersion in hot and cold water, which is called contrast water therapy (CWT). CWT may cover the entire body or a selected part of it. Despite numerous studies, a consistent methodology for using this regenerative therapy is lacking (Bieuzen et al., 2013). As described in scientific literature, partial immersion in hot water (HWI) or cold water (CWI) most often involves several cycles with a total time of 6–20 min (Wang et al., 2022); the water temperature ranges from 3°C to 15°C for a cold stimulus and 40°C–45°C for a warm stimulus (Wang et al., 2022). Recently, Game Ready (GRT) (Avanos Medical, United States, 2020) - a new portable equipment for contrast therapy- has been gaining popularity (Alexander et al., 2021). It can be used as a local monotherapy with heat or cold and also as contrast therapy (Allan et al., 2022; Dupont et al., 2017). GRT combines an alternating hot and cold stimulus applied to a specific tissue with a pressure cuff. The pressure can be modulated in the range of 15–75 mmHg, and the temperature from 3°C to 45°C, while the duration can be regulated from 10 to 30 min (Alexander et al., 2021; Priego-Quesada et al., 2021).

Some results suggested introducing compression into cold or heat therapy to increase the effectiveness of the recovery (Allan et al., 2022; El-Sheikh et al., 2022). However, not all research results confirm the effectiveness of compression (Valenzuela et al., 2018). Consequently, the knowledge of comparing CWT and GRT therapy is lacking. Although the mechanisms explaining the effects of contrast therapy remain unclear (Trybulski et al., 2024d), in the scientific literature, we can find evidence of the effectiveness of this therapy in terms of reducing delayed muscle soreness syndrome (Malanga et al., 2015), reducing muscle tone and improving muscle elasticity (Trybulski et al., 2024d) and decrease muscle stiffness (Huxel et al., 2008). In addition, it has a beneficial effect on tissue perfusion (Cezar et al., 2016; Trybulski et al., 2024a), improvement of muscle strength and power (Dupont et al., 2017), reduction of muscle pain (Wang et al., 2022) acceleration of the removal of inflammatory factors (Malanga et al., 2015), reduction of swelling (Vaile et al., 2007), changes in tissue temperature (Medeiros et al., 2022) and hormonal changes (Wang et al., 2022). Despite many beneficial effects, not all research results confirm the effectiveness of contrast therapy (S. D. French et al., 2006). In addition, many factors which may affect the effectiveness of treatment are described in the literature: the thickness of the subcutaneous tissue (Petrofsky et al., 2015), individual responses of the sympathetic system to heat and cold stimuli (Wang et al., 2022) and favoring therapy (Goswami et al., 2022).

Our previous research on the effects of GRT on quadriceps femoris muscle in MMA athletes showed that it lowers T, E, and S immediately after use (post 5min) and post 1 h compared to the rest values. Simultaneously, it increases perfusion and tissue temperature immediately after use (post 5 min) and post 1 h compared to rest values. In that study, GRT showed no effect on PPT. The effects were measured without fatigue and compared to the sham therapy (Trybulski et al., 2024c). Another of our studies on the forearm muscles of MMA fighters showed that GRT increases tissue perfusion and decreases T and S when comparing immediate post-fatigue to post-therapy 5 min results (Trybulski et al., 2024b). Despite both therapies being widely used, the literature lacks a comparison of the effects of GRT and CWT in combat sports athletes.

Our study aimed to compare the immediate effects of compression contrast heat and cold therapy (GRT) and water immersion heat and cold therapy (CWT) on forearms’ muscles’ stiffness, tone, flexibility, pressure pain threshold, and maximal flexors force in martial arts athletes. Comparing the effectiveness of these forms of contrast therapy may become a direction for future research on assessing the usefulness of contrast therapy in post-exercise muscle regeneration.

## Material and methods

### Participants

Thirty young, healthy male volunteers, amateur combat sports competitors (Mixed Martial Arts, Judo, Brazilian Jiu Jitsu) (age: 28.20 ± 7.57 years, BMI: 26.35 ± 4.06 [kg/m^2^] training experience: 10.37 ± 7.34 years) (Table 1) were randomly selected according to the following inclusion criteria: age 18–40 years; minimum 3 years of experience in combat sports training; training at least four times a week. Considering McKay’s participant classification scheme, the group of competitors belonged to Tier 2, 3: Highly Trained/National Level (McKay et al., 2022). The body characteristics of the participants are divided into experimental and control groups, and their comparison is presented in Table 1. Study exclusions included elevated pre-test blood pressure (blood pressure > 140/90 mm Hg); currently treated injuries, damaged skin, or unspecified skin lesions at the measurement sites; having a tattoo at the measurement area; taking any medications, including painkillers. Exclusions were also made in the event of extreme fatigue, fever, infection, or at the explicit request of the participant (Holwerda et al., 2013). Written informed consent was obtained from participants after they were informed about the study’s aims, risks, and conditions. Participants were required to refrain from training for 24 h before the tests. Additionally, due to tissue perfusion measurements (these results were not presented in this article), participants were asked to refrain from consuming any ergogenic drinks (i.e., coffee, tea, energy drinks, etc., a list of excluded products was provided to participants) minimum 24 h before the examination (Trybulski et al., 2024a). Exclusion from the study could occur at any time during its duration at the participant’s request. The authors declare that they have the consent of the participant in the photo to use the image in a scientific article. Before the study, each participant completed a health questionnaire and informed consent.

**TABLE 1:** Characteristics of research samples, student’s t-test for independent samples.

Variable	Experimental group (n = 15)				Control group (n = 15)				t	p
	M	SD	95% CI	95% CI	M	SD	95% CI	95% CI		
Age	28.20	7.57	24.01	32.39	28.40	7.93	24.01	32.79	−0.071	0.94
Weight [kg]	86.13	17.09	76.67	95.60	86.60	16.25	77.60	95.60	−0.077	0.94
Height [cm]	180.27	6.05	176.91	183.62	180.80	5.76	177.61	183.99	−0.25	0.81
Training experience (years)	10.37	7.34	6.30	14.43	10.47	6.94	6.63	14.31	−0.038	0.97
Body Mass Index [kg/m^2^]	26.34	4.07	24.09	28.58	26.38	3.86	24.24	28.52	−0.001	0.99

No significant differences were found between the results of body structure parameters and training experience for the analyzed groups, p > 0.05.

### Study design

In this single-blind, randomized clinical trial, 30 participants were randomly assigned to control and experimental groups. Group allocation was achieved by simple 1:1 randomization using a random sequence generated on the randomizer.org website. The randomization process was independent of treatment duration and study personnel. The order of examining therapies (GRT or CWT) was randomly chosen by coin toss.

**FIGURE 1:**
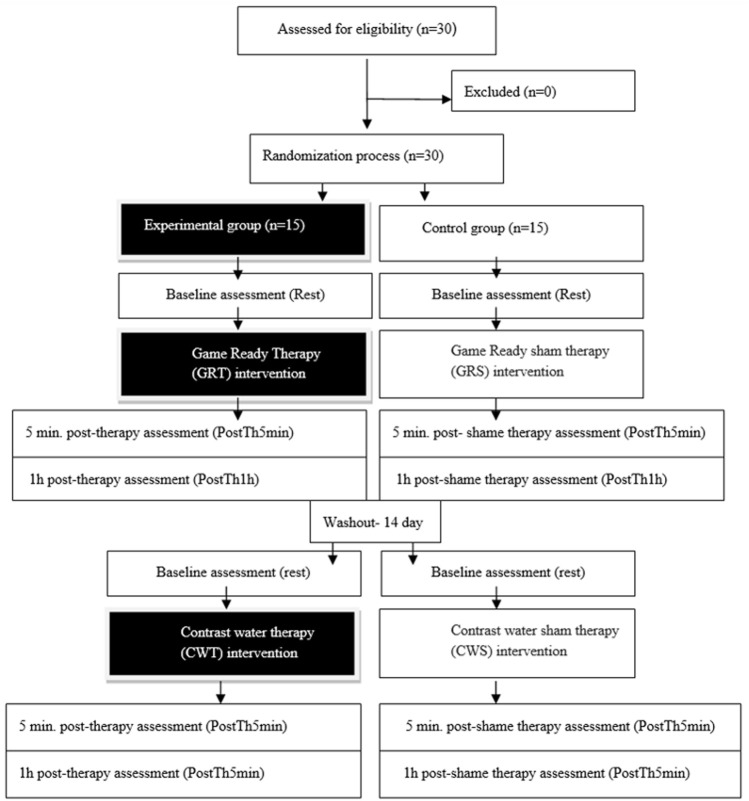
Study design.

In the first phase, the experimental group (n = 15) was subjected to 10 min of GRT, and the control group (n = 15) to 10 min of GRS. After a 2-week break, in the second phase, the experimental group was subjected to 10 min of CWT, and the control group was subjected to 10 min of sham water therapy (CWS). Interventions of experimental contrast therapy and sham therapy were performed according to the previous study methodology (Trybulski et al., 2024d). The study design is presented in Figure 1. The study was approved by the ethics committee of the National Council of Physiotherapists (No. 9/22 of 6 April 2022) and registered in the clinical trials register under the number https://doi.org/10.1186/ISRCTN90040217 and conducted in accordance with the Declaration of Helsinki.

### Interventions

#### Warm, cold, and pressure contrast therapy protocol

**FIGURE 2:**
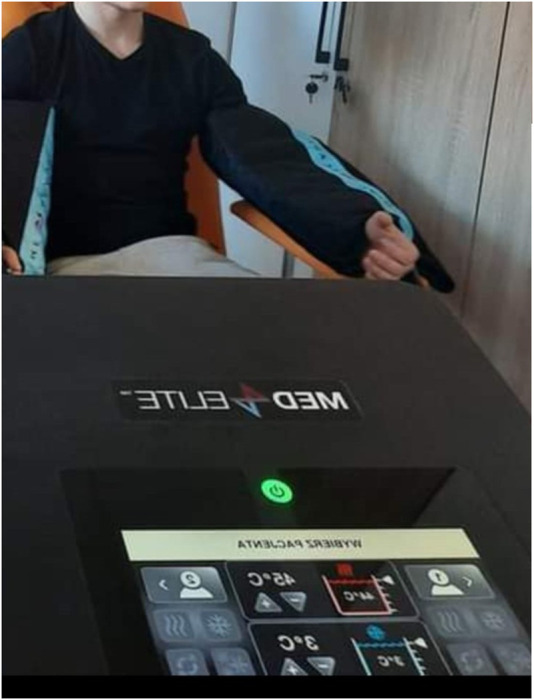
Game Ready contrast pressure therapy device.

Experimental (therapeutic interventions - GRT) used a Game Ready device (MED4 ELITE, Avanos Medical, United States, 2020) with a cuff placed on the forearm, which provided alternating stimulation for 1 minute with cold at a temperature of 3°C and a pressure of 75 mmHg (10 kPa), and then 1 minute with heat at 45°C and compression of 15 mmHg (3.33 kPa). The total treatment time was 10 min. The control group was receiving sham therapy (GRS), and the same procedure was followed, including 1 min of cold and 1 min of warm stimulus for 10 min. The temperature was 15°C for the cold stimulus (lowest stimulus possible GR regulation) and pressure of 15 mmHg (lowest stimulus possible GR regulation) and 36°C for the warm stimulus (neutral stimulus) at a pressure of 15 mmHg (Trybulski et al., 2024a). The selected parameters for the control group were the least intense and for the experimental group, the most intense possible to produce the most significant effect, based on the assumption that the body’s reaction is proportional to the applied stimulus, i.e., within the safe range for the participants. An image documenting the intervention is shown in Figure 2.

#### Warm and cold-water contrast therapy protocol

**FIGURE 3:**
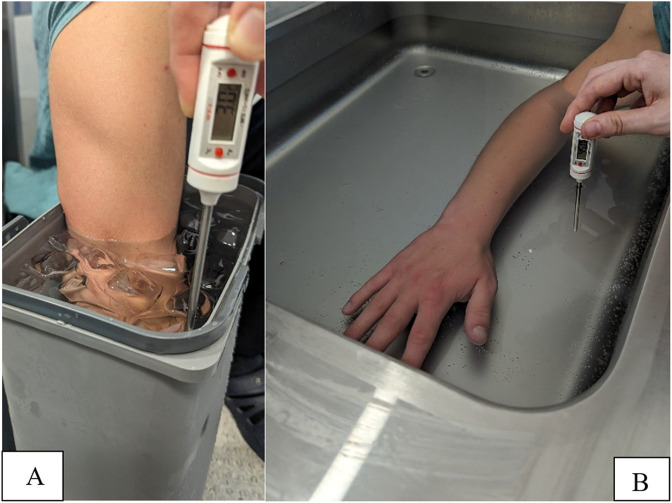
Partial contrast water immersion therapy: **(A)** cold water, **(B)** hot water.

The experimental therapy consisted of immersing the forearm for 10 min, alternately every minute, in a bucket of water and ice at a temperature of 3°C and in warm water heated by a device with a thermostat to a temperature of 45°C (Bemar, 2020, Poland) so that the stimulus temperatures for the GRT and CWT experimental groups were the same. In the control group, sham therapy was used for the same time as in the experimental group: immersion for 10 min, alternating every minute in water at a temperature of 15°C and 36°C. Cold and hot water temperatures were controlled with a thermometer, and warm water was controlled with a thermostat (Figure 3).

### Measurements

**FIGURE 4:**
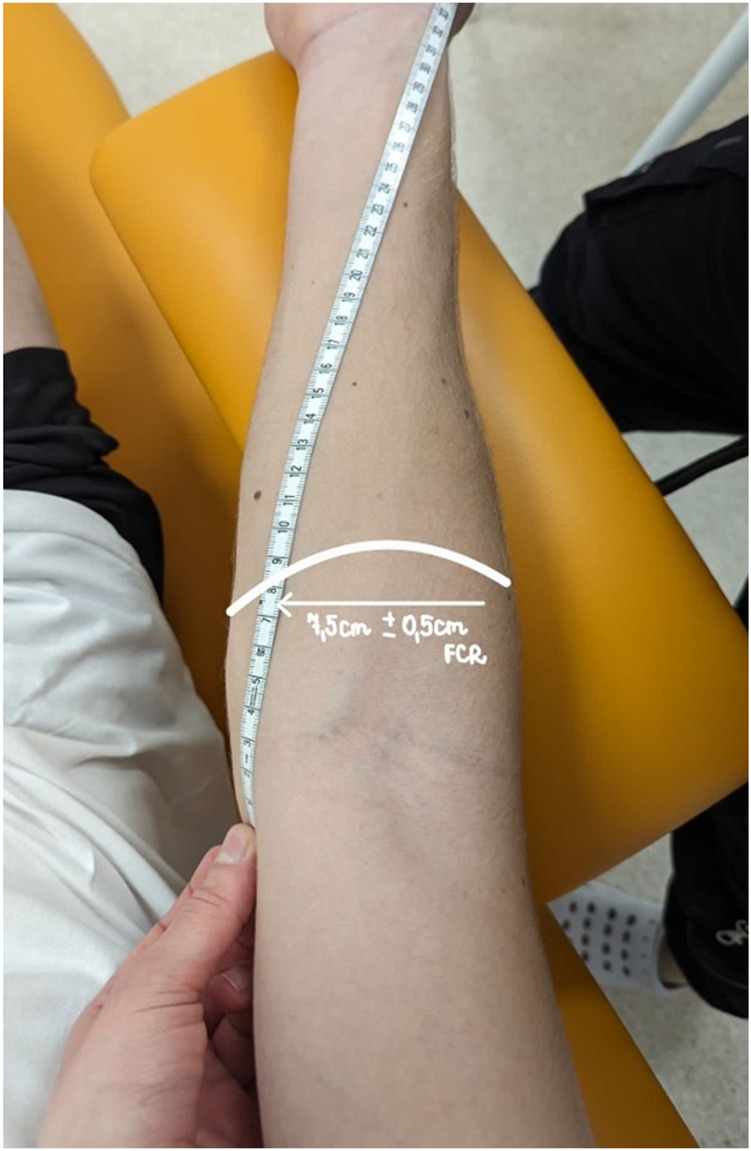
Marking the measurement point for FCR.

Using an ultrasonic device (USG - SONOSCAPE P20, China, 2021), the broadest cross-section of the flexor carpi radialis (FCR) muscle was determined and the measuring place was marked with a marker (Trybulski et al., 2024a). A line was drawn connecting the styloid process with the medial epicondyle of the humerus, defining a perpendicular line extending within 7 cm from the epicondyle (7.5 ± 0.5 cm) (Iivarinen et al., 2011) (Figure 4). All measurements were made at the same point of the dominant hand. During the measurements, participants were in a relaxed sitting position on a chair with the same elbow bending angles (approximately 70°) (Zebrowska et al., 2019). The following measurements were performed in all participants: (I) muscle tone (T - [Hz]), (II) dynamic stiffness (S - [N/m]), (III) elasticity (E - [arb - relative arbitrary unit]), (IV) pressure pain threshold (PPT - [N/cm^2^]), (V) isometric muscle strength - Fmax - [kgf]. Measurements were made in 3 periods: (I) at Rest (Rest), (II) 5 minutes after therapy (PostTh5min), and 1 hour after therapy (III) (PostTh1h). All study participants were examined under the same time conditions (10:00 to 12:00) at the Provita Medical Center, where the same temperature conditions prevailed (21°C). The same trained physiotherapists performed all measurements.

#### Myotonometry

Measurements were taken with a myotonometer MyotonPRO (Myoton Ltd, Estonia, 2021). MyotonPRO is a digital device consisting of a device body and a depth probe (Ø 3 mm). Through the probe, a pre-pressure (0.18 N) is applied to the surface, which compresses the underlying material. A mechanical impulse (0.4 N, 15 ms) is then released through the device, which deforms the medium quickly (Trybulski et al., 2024a). Myotonometry is a reliable measurement method and can detect differences in physical properties compared to stretched muscle fibers (Bartsch et al., 2023; Melo et al., 2022). The measurement method consists of registering the damped natural vibrations of soft biological tissue in the form of an acceleration signal and then simultaneously calculating the parameters of the state of stress and biomechanical properties: muscle tone (T – [Hz]), stiffness (S – [N/m]), and elasticity (E - [arb- relative arbitrary unit]) (Bartsch et al., 2023). Stiffness assessed using myotometry is based on the theory of free oscillation and results from the natural oscillation of tissues in response to short-term mechanical exposure of the skin (Trybulski et al., 2024a). Tissues can also recover their original shape after deformation. This property, measured in this study, is called elasticity (E). The higher the elasticity, the faster the tissue returns to its original shape (Feng et al., 2018). Three measurements were taken, and then average values were calculated and processed in the statistical analysis.

#### Pressure pain threshold

Pressure pain threshold (PPT - [N/cm^2^]) was measured using an FPIX algometer (Wagner Instruments, Greenwich, CT, United States, 2013). Determining the PPT is an attempt to assess this pain parameter objectively and is characterized by high repeatability and reliability (Park et al., 2011). Participants were subjected to a compression test with a probe three times (probe radius, r = 4 mm), causing compressive forces in a specific marked area that did not change during the test. The pressure value [N/cm^2^] was digitally displayed on the screen and calculated as the average of 3 measurements. The pressure was applied until the test stimulus was signaled as unpleasant (Trybulski et al., 2024a).

#### Isometric muscle force

An electronic hand-held dynamometer (EH106 China, 2020) measured maximum forearm muscle force (Fmax). The participant performing a try stood with the arms hanging freely (Folhes et al., 2022). On this basis, the maximum strength of the forearm muscles was calculated and expressed in [kgf]. The grip strength test consisted of the maximum 5-s contraction of the forearm muscles while squeezing the dynamometer. The method is characterized by high repeatability and reliability (Venegas-Carro et al., 2022). Before the test, each participant warmed up by exerting maximum pressure on a small ball ten times and then stretching the forearm muscles for 10 s (Trybulski et al., 2024a).

### Statistical methods

Data are presented as means and SD. Shapiro-Wilk, Levene, and Mauchly’s tests were used to test sample data variance’s normality, homogeneity, and sphericity, respectively. For significance analysis of the follow-up measures (post 5min and post 1 h) the two-way functional repeated measures analysis of variance was used. Effect sizes for main effects and interactions were determined by partial eta squared (η_p_^2^). Partial eta-squared values were classified as small (0.01–0.059), moderate (0.06–0.137), and large (>0.137). Post hoc comparisons were performed using the Bonferroni test to locate differences between mean values when a main effect or interaction was found. Percentage changes with 95% confidence intervals (95% CI) were also calculated. Statistical significance was set at p < 0.05. For pairwise comparisons, effect sizes were determined by Cohen’s d and were characterized as large (d > 0.8), moderate (d between 0.79 and 0.5), small (d between 0.49 and 0.20), and trivial (d < 0.2) (Cohen, 1992). Statistical significance was set at p < 0.05. The repeated measure ANOVA within-between interactions with an effect size of at least 0.25, α = 0.05, and 1-β = 0.95 gave a statistical power of 97.37% and a minimum sample size of 10 subjects. All statistical analyses were performed using Statistica 13.1 (Statsoft, Inc., Tulsa, OK, United States)

## Results

The two samples were in the same condition prior to the protocol (Table 1). The results of each intervention are presented in Table 2.

**TABLE 2:** Comparisons between the experimental (GRT and CWT) and control groups (GRS and CWS) for all assessment variables during Rest and Post-Th periods (5 min and 1 h after).

Variable	Assessment period	Experimental group (n = 15) GRT	Control group (n = 15) GRS	Experimental group (n = 15) CWT	Control group (n = 15) CWS
		M ± SD (−95%–95%) CI	M ± SD (−95%–95%) CI	M ± SD (−95%–95%) CI	M ± SD (−95%–95%) CI
Muscle tone [Hz]	Rest	*** ^,###^19.40 ± 0.68 (19.02–19.78)	19.11 ± 0.59 (18.78–19.44)	18.54 ± 1.55 (17.68–19.40)	18.78 ± 2.12 (17.61–19.95)
	PostTh5min	***16.19 ± 0.86***^, #, $$$^(15.71–16.66)	19.07 ± 0.55*** (18.76–19.37)	^18.28 ± 2.97^#^(16.64–19.92)	^19.32 ± 2.48^$$$^(17.95–20.70)
	PostTh1h	^###^16.97 ± 0.63 (16.62–17.31)	18.99 ± 0.53 (18.69–19.28)	18.26 ± 1.68 (17.33–19.19)	19.38 ± 2.02 (18.26–20.50)
Elasticity [arb]	Rest	***^, ###^284.73 ± 21.45 (272.85–296.61)	281.60 ± 17.12 (272.12–291.08)	***^, ###^348.00 ± 24.67 (334.34–361.66)	323.87 ± 41.17 (301.07–346.66)
	PostTh5min	***244.93 ± 15.10 (236.57–253.29)	282.87 ± 19.16 (272.26–293.48)	***307.40 ± 36.54 (287.17–327.63)	323.87 ± 40.80 (301.27–346.46)
	PostTh1h	^###^259.20 ± 19.16 (248.59–269.81)	280.73 ± 18.41 (270.54–290.93)	^###^320.20 ± 38.18 (299.06–341.34)	328.20 ± 57.14 (296.56–359.84)
Stiffness [N/m]	Rest	***^, ###^1.38 ± 0.08 (1.33–1.42)	1.36 ± 0.07 (1.32–1.40)	***^, ###^1.38 ± 0.08 (1.33–1.42)	1.36 ± 0.07 (1.33–1.40)
	PostTh5min	***1.19 ± 0.07 (1.15–1.23)	1.36 ± 0.09 (1.31–1.41)	***1.19 ± 0.07 (1.16–1.23)	1.36 ± 0.09 (1.31–1.41)
	PostTh1h	^###^1.23 ± 0.04 (1.20–1.25)	1.38 ± 0.09 (1.33–1.43)	^###^1.23 ± 0.04 (1.21–1.25)	1.38 ± 0.09 (1.33–1.43)
PPT [N/cm^2^]	Rest	***^, ###^84.30 ± 7.58 (80.10–88.50)	84.32 ± 8.57 (79.57–89.07)	***^, ###^84.30 ± 7.58 (80.10–88.50)	84.32 ± 8.57 (79.57–89.07)
	PostTh5min	***91.32 ± 7.35 (87.25–95.39)	84.20 ± 7.21 (80.21–88.19)	***91.32 ± 7.35 (87.25–95.39)	84.20 ± 7.21 (80.21–88.19)
	PostTh1h	^###^92.13 ± 7.42 (88.02–96.24)	84.50 ± 7.49 (80.35–88.65)	^###^92.13 ± 7.42 (88.02–96.24)	84.50 ± 7.49 (80.35–88.65)
Fmax [kgf]	Rest	***^, ###^49.43 ± 4.78 (46.79–52.08)	43.89 ± 2.76 (42.36–45.42)	***^, ###^51.11 ± 5.42 (48.11–54.11)	43.80 ± 2.72 (42.29–45.31)
	PostTh5min	***53.52 ± 5.14 (50.67–56.37)	44.09 ± 2.90 (42.48–45.69)	***53.65 ± 5.15 (50.80–56.51)	44.27 ± 3.01 (42.61–45.94)
	PostTh1h	^###^52.87 ± 5.56 (49.79–55.95)	43.94 ± 3.40 (42.06–45.82)	^###^52.88 ± 5.69 (49.73–56.03)	44.20 ± 3.57 (42.22–46.18)

Statistically significant differences between Rest - PostTh5min - PostTh1h were marked before the number. Statistically significant differences between Experimental and Control were marked after the number. Number of markers mean level of significance:***^/###/$$$/^^^^p < 0.001;**^/##/$$/^^^p < 0.01;*^/#/$/^^p < 0.05.All significances of differences are reported from post-hoc tests for interactions.

**FIGURE 5:**
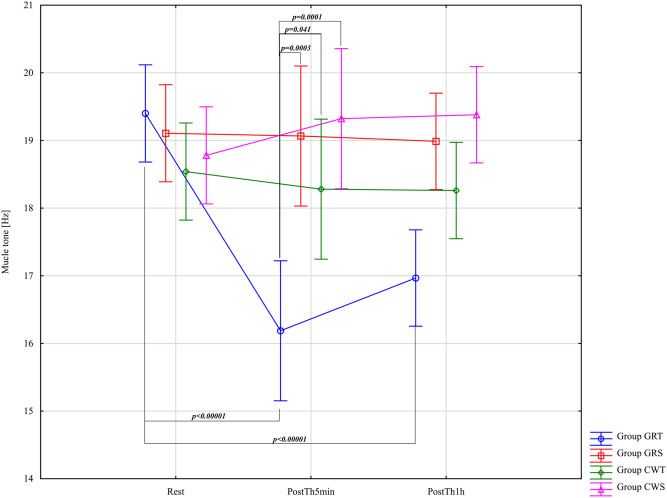
Main effects of muscle tone (T [Hz]) for groups in the assessment period: Rest and PostTh 5 min and 1 h. GRT, experimental group, Game Ready Therapy; GRS, control group, Game Ready sham therapy; CWT, experimental group, contrast water therapy; CWS, control group, contrast water sham therapy. All significances of differences are reported from post-hoc tests for interactions.

The results of the analysis of variance for muscle tone (T - [Hz]) allowed for the finding of significant differences for the main effects: Group F = 4.19; (p = 0.01); η_p_^2^ = 0.18; Before –5min-1 h after F = 10.90; (p = 0.00005); η_p_^2^ = 0.16 and for the interaction group* Before –5min-1 h after F = 14.26; (p < 0.00001); η_p_^2^ = 0.43. Results post-hoc analysis performed after an interaction effect in a two-way ANOVA for GRT group - significant differences were found between Rest (M = 19.40 ± 0.68) and PostTh5min (M = 16.19 ± 0.86) (p < 0.00001), as well as between Rest and PostTh1h (M = 16.97 ± 0.63; p < 0.00001). No significant difference was observed between PostTh5min and PostTh1h (p = 0.99). Thus, both PostTh5min and PostTh1h values were significantly lower than at Rest. The results post-hoc analysis performed after an interaction effect in a two-way ANOVA in the GRT PostTh5min group M = 16.19 ± 0.86 were significantly lower than in the GRS control group PostTh5min M = 19.07 ± 0.55; (p = 0.0003; d = 3.99) and then in the experimental group CWT PostTh5min (M = 18.28 ± 2.97; p = 0.041; d = 0.96) and in the CWS control group (CWS for CWT) PostTh5min (M = 19.32 ± 2.48; p = 0.0001; d = 1.69). Muscle Tone [Hz] results in the GRT group for PostTh5min were significantly lower than in the GRS, CWT, and CWS groups. These results are presented in Figure 5.

**FIGURE 6:**
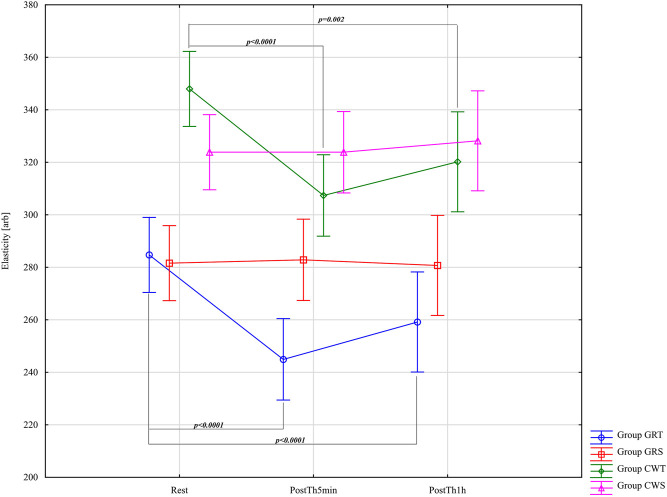
Main effects of elasticity (E [arb]) for groups in the assessment period: Rest and PostTh 5 min and 1 h. GRT, experimental group, Game Ready Therapy; GRS, control group, Game Ready sham therapy; CWT, experimental group, contrast water therapy; CWS, control group, contrast water sham therapy. All significances of differences are reported from post-hoc tests for interactions.

The results of the analysis of variance for Elasticity - E-[arb] allowed for the finding of significant differences for the main effects: Group F = 18.44; p < 0.0001; η_p_^2^ = 0.50; Before –5min-1 h after F = 20.05; p < 0.0001; η_p_^2^ = 0.26 and for the interaction group* Before –5min-1 h after F = 7.43; p < 0.0001; η_p_^2^ = 0.28. Results post-hoc analysis performed after an interaction effect in a two-way ANOVA for GRT Group - significant differences were observed between Rest (M = 284.73 ± 21.45) and PostTh5min (M = 244.93 ± 15.10) (p < 0.0001; d = 2.15, large effect), and between Rest and PostTh1h (M = 259.20 ± 19.16; p < 0.0001; d = 1.26, large effect). No significant difference was found between PostTh5min and PostTh1h (p = 0.99). In other words, the values at PostTh5min and PostTh1h were significantly lower than at Rest. No significant differences were detected in the GRS control group (p > 0.05). Results post-hoc analysis performed after an interaction effect in a two-way ANOVA for CWT Group - significant differences were noted between Rest (M = 348.00 ± 24.67) and PostTh5min (M = 307.40 ± 36.54; p < 0.0001; d = 1.32, large effect), as well as between Rest and PostTh1h (M = 320.20 ± 38.18; p = 0.002; d = 0.86, large effect). No significant difference was observed between PostTh5min and PostTh1h (p = 0.99). Thus, PostTh5min and PostTh1h values were significantly lower than at Rest. No significant differences were found in the CWS control group (p > 0.05). The effect size was large across both experimental groups (GRT and CWT). However, the GRT group (referred to here as GRS) showed a higher effect size than the CWT group at Rest, PostTh5min, and PostTh1h. These results are presented in Figure 6.

**FIGURE 7:**
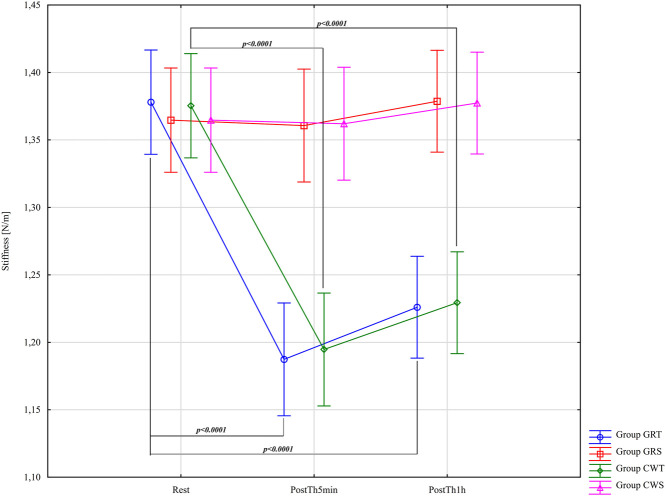
Main effects of Stiffness (S – [N/m]) for groups in the assessment period: Rest and PostTh 5 min and 1 h. GRT, experimental group, Game Ready Therapy; GRS, control group, Game Ready sham therapy; CWT, experimental group, contrast water therapy; CWS, control group, contrast water sham therapy.

Results of the analysis of variance for Stiffness (S – [N/m]), subject to the application of a detailed difference criterion for main effects: Group F = 11.52; (p < 0.0001); η_p_^2^ = 0.38; Before –5min-1 h after F = 78.11; (p < 0.0001); η_p_^2^ = 0.58 and for the group* Before –5min-1 h after F = 27.46; (p < 0.0001); η_p_^2^ = 0.60. Results post-hoc analysis performed after an interaction effect in a two-way ANOVA for GRT Group - significant differences were found between Rest (M = 1.38 ± 0.08) and PostTh5min (M = 1.19 ± 0.07; p < 0.0001; d = 2.53, large effect), as well as between Rest and PostTh1h (M = 1.23 ± 0.04; p < 0.0001; d = 2.37, large effect). No significant difference was observed between PostTh5min and PostTh1h (p = 0.97). Thus, both PostTh5min and PostTh1h values were significantly lower than at Rest. No significant differences were detected in the GRS control group (p > 0.05). Results post-hoc analysis performed after an interaction effect in a two-way ANOVA for CWT Group - similarly, significant differences were noted between Rest (M = 1.38 ± 0.08) and PostTh5min (M = 1.19 ± 0.07: p < 0.0001; d = 2.53, large effect), and between Rest and PostTh1h (M = 1.23 ± 0.04; p = 0.0001; d = 2.37; large effect). No significant difference was found between PostTh5min and PostTh1h (p = 0.99). As in the GRT group, PostTh5min and PostTh1h values were significantly lower than at Rest. No significant differences were observed in the CWS control group (p > 0.05). In both experimental groups (GRT and CWT), Cohen’s d indicated a very large effect, and the magnitude of these effects was the same in both interventions. These results are presented in Figure 7.

**FIGURE 8:**
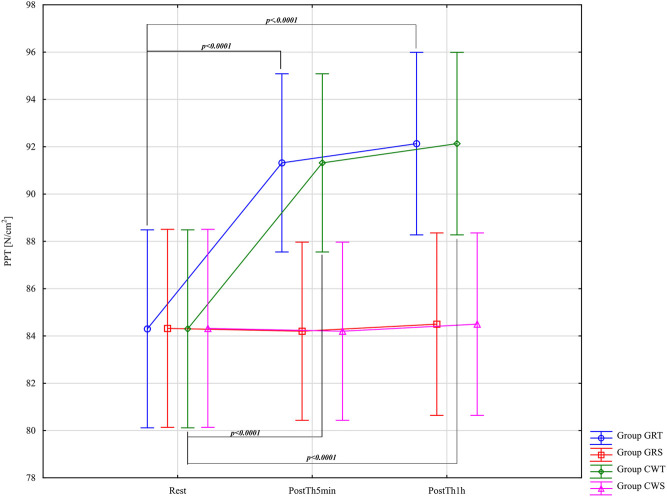
Main effects of pressure pain threshold (PPT - [N/cm^2^]) for groups in the assessment period: Rest and PostTh 5 min and 1 h. GRT, experimental group, Game Ready Therapy; GRS, control group, Game Ready sham therapy; CWT, experimental group, contrast water therapy; CWS, control group, contrast water sham therapy. All significances of differences are reported from post-hoc tests for interactions.

The results of the analysis of variance for pressure pain threshold (PPT - [N/cm^2^]) did not identify significant differences for the main effects Group F = 2.19; (p = 0.1) η_p_^2^ = 0.11. The results of the analysis of variance for PPT allowed for the finding of significant differences only for the main effect: Before -5min-1 h after F = 61.78; (p < 0.0001); η_p_^2^ = 0.52 and for the interaction group* Before –5min-1 h after F = 20.00; (p < 0.0001); *η*_p_^2^ = 0.52. Results post-hoc analysis performed after an interaction effect in a two-way ANOVA for GRT Group - significant differences were found between Rest (M = 84.30 ± 7.58) and PostTh5min (M = 91.32 ± 7.35; p < 0.0001; d = 0.94; large effect), and between Rest and PostTh1h M = 92.13 ± 7.42; p < 0.0001; d = 1.04; large effect). No significant difference was observed between PostTh5min and PostTh1h (p = 0.99). Therefore, PostTh5min and PostTh1h values were significantly higher than at Rest. No significant differences were noted in the GRS control group (p > 0.05). Results post-hoc analysis performed after an interaction effect in a two-way ANOVA for CWT Group - significant differences were observed between Rest (M = 84.30 ± 7.58) and PostTh5min (M = 91.32 ± 7.35; p < 0.0001; d = 0.94; large effect), as well as between Rest and PostTh1h (M = 92.13 ± 7.42; p < 0.0001; d = 1.04; large effect). No significant difference was found between PostTh5min and PostTh1h (p = 0.99). Thus, PostTh5min and PostTh1h values were significantly higher than at Rest. There were no significant differences in the CWS control group (p > 0.05). In both experimental groups (GRT and CWT), Cohen’s d values were large and of the same magnitude, indicating similarly strong effects. These results are presented in Figure 8.

**FIGURE 9:**
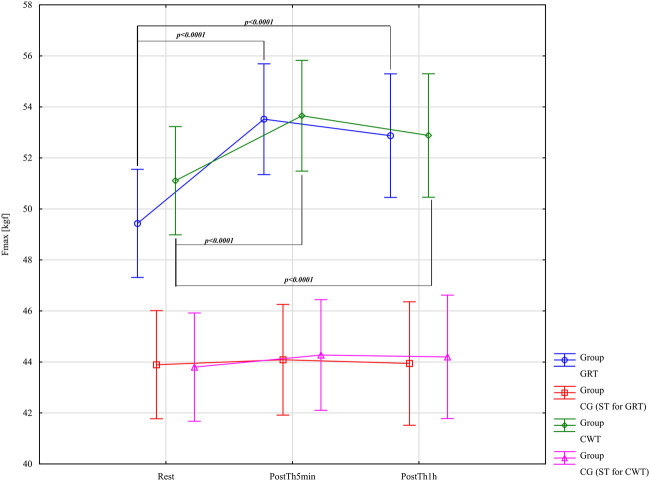
Main effects of isometric muscle force - Fmax - [kgf] for groups in the assessment period: Rest and PostTh 5 min and 1. GRT, experimental group, Game Ready Therapy; GRS, control group, Game Ready sham therapy; CWT, experimental group, contrast water therapy; CWS, control group, contrast water sham therapy. All significances of differences are reported from post-hoc tests for interactions.

The results of the analysis of variance for Fmax [kgf] allowed for the identification of significant differences for the main effects: Group F = 18.60; (p < 0.0001); η_p_^2^ = 0.50; Before –5min-1 h after F = 61.85; (p < 0.0001); η_p_^2^ = 0.52 and for the interaction group* Before –5min-1 h after F = 16.52; (p < 0.0001); η_p_^2^ = 0.47. Results post-hoc analysis performed after an interaction effect in a two-way ANOVA for GRT Group - significant differences were found between Rest (M = 49.43 ± 4.78) and PostTh5min (M = 53.52 ± 5.14; p < 0.0001; d = 0.82; large effect), as well as between Rest and PostTh1h (M = 52.87 ± 5.56; p < 0.0001; d = 0.66; moderate effect). No significant difference was observed between PostTh5min and PostTh1h (p = 0.97). Therefore, both PostTh5min and PostTh1h values were significantly higher than at Rest. No significant differences were found in the GRS control group (p > 0.05). Results post-hoc analysis performed after an interaction effect in a two-way ANOVA for CWT Group - significant differences were observed between Rest (M = 51.11 ± 5.42) and PostTh5min (M = 53.65 ± 5.15; p < 0.0001; d = 0.48; small effect), as well as between Rest and PostTh1h (M = 52.88 ± 5.69; p < 0.0001; d = 0.32; small effect). No significant difference was found between PostTh5min and PostTh1h (p = 0.99). Thus, PostTh5min and PostTh1h values were significantly higher than at Rest. Comparison of Effect Sizes - the GRT experimental group showed larger effect sizes (large/moderate) compared to the CWT experimental group (small effect). In the CWS control group, no significant differences were detected (p > 0.05). These results are presented in Figure 9.

## Discussion

The primary objective of this study was to evaluate the immediate effects of short-term use of two forms of contrast therapy (play-ready contrast compression therapy and contrast water immersion) on specific muscle biomechanical properties such as muscle tone, muscle stiffness and elasticity, pressure pain threshold, and isometric muscle strength. The main findings of the study confirm the positive impact of both contrast therapy methods on the measured variables, however, no significant differences were found between the GRT and CWT experimental groups. The Cohen effects were similar. Only in the assessment of muscle tone reduction, statistically significant results were found for the GRT group compared to the CWT group. These results help optimize the contrast therapy protocol and provide guidance for future sports recovery research.

In the present study we focused on stiffness, muscle tone and elasticity as crucial parameters influencing muscle strength, power generation and the risk of injury in sports. It is essential to highlight that for proper energy dissipation, the muscle bundles must actively elongate, maintaining the right balance of elasticity and stiffness (Nuñez et al., 2022; Roberts and Konow, 2013). Studies conducted both *in situ* and *in vivo* indicate that the tendon fibers surrounding the muscles can delay this elongation during energy-dissipating events by temporarily absorbing impact energy and then releasing it to work on the muscle bundles. This intricate mechanism relies not only on appropriate neural control but also on adequate blood distribution through microcirculation (Bieuzen et al., 2013; Cochrane, 2004).

Muscle tone, elasticity, and stiffness are mainly maintained through the complex interplay of spinal and supraspinal mechanisms, the disruption of which can cause many changes in the biomechanical properties of muscles (Graven-Nielsen et al., 2002). It is widely accepted that a thermal stimulus that causes an increase in muscle hyperemia reduces muscle tone and improves muscle stiffness and elasticity (Kim et al., 2020b). Although these mechanisms are not clear, non-myogenic regulation of muscle tone associated with increased perfusion is also accepted (Trybulski et al., 2024a). Elevated Ca^2+^ concentrations in the cytosol, which occur under hypoxia resulting from impaired perfusion, cause muscle contraction by activating phosphorylation of myosin light chains and subsequent actomyosin cross-bridging, which generates an increase in tone (Kim et al., 2020b). Activation of the capillary system, which eliminates subclinical manifestations of tissue hypoxia, can therefore reduce muscle tone (Zebrowska et al., 2019).

The effects of contrast therapy on biomechanical parameters occur as thermal, mechanical and chemical effects, alone or as mixed effects (Moore et al., 2023). The hydrostatic pressure of water and the pneumatic cuff is associated with several changes, including fluid shift from the extracellular to the intravascular space, which may aid the elimination process of metabolic products (Wiśniowski et al., 2022). Although definitive conclusions cannot be drawn due to the many limitations of these studies, the observed effects may indicate that GRT and CWT therapy may change muscle biomechanical properties over time (Zebrowska et al., 2019).

The results of our study showed a similar effect of forms of contrast therapy on reducing muscle pain and support the general hypothesis of the analgesic effect of contrast therapy (Colantuono et al., 2023). Although pain is one of the most frequently analyzed variables in the scientific literature, there is still insufficient evidence for the use of contrast therapy (especially game-ready) in the treatment of muscle pain. Physical therapies for the modulation of muscle pain are common practice among clinicians (Wang et al., 2022). Physical methods, including contrast therapy, may affect the peripheral, spinal or supraspinal level of the pain pathway, depending on the type, intensity, duration and location of the stimuli (D. N. French et al., 2008). Therapy contras may activate thermosensitive TRP (transient receptor potential) channels that modulate peripheral and central thermal sensitivity (Brederson et al., 2013). Neuroimaging studies show that heat can activate endogenous opioid and cortical serotonergic systems (Quittan, 2002). This is achieved by improving the connectivity between the thalamus anterior cingulate cortex and periaqueductal gray. Recent evidence suggests that heat therapy reduces central markers of sensitization, including neuroinflammation and NMDA (N-methyl-D-aspartate receptor) receptor activation (Goswami et al., 2022).

In the literature contrast water therapy has been shown to eliminate the adverse effects of exercise-related muscle damage (EAMD), inflammation and delayed onset muscle soreness (DOMS) (Bieuzen et al., 2013), while increasing the rate of muscle strength recovery (Colantuono et al., 2023) joint power and mobility after strenuous exercise (S. D. French et al., 2006). Colantuono et al. (2023) note that previous studies of contrast therapy combined with compression have not analyzed the ability of muscle recovery after intense exercise or assessed the recovery of intramuscular glycogen stores (Colantuono et al., 2023). The authors’ results are intriguing and suggest a positive effect of contrast therapy on recovery from intense eccentric exercise and the regeneration of intramuscular glycogen stores associated with EAMD (Versey et al., 2012). In the scientific literature, the results of the impact of various forms of contrast therapy on muscle regeneration could be clearer. Yoshida et al. (2022) suggest that heat or cold medicine in the first 30 min after intense eccentric exercise is not sufficient to prevent DOMS (Yoshida et al., 2022). The authors mentioned before used different protocols and stimuli from those used in the presented study. This may be related to the lack of compression during alternating hot and cold stimulation. Individualization of contras therapy is equal to maximizing the analgesic effect while ensuring safety and tolerability. The treatment plan should include application based on the type of pain, impact on the patient, and administration of temperature changes. Factors such as comorbidities, age, gender and individual variability in response to endogenous analgesics may influence results (Petrofsky et al., 2015). Over time, monitoring and feedback can help identify hidden protocols that may be used to treat post-exertional muscle pain.

There is insufficient evidence regarding the effect of contrast therapy on improving muscle strength and power. In our study, the impact of both forms of treatment improved the results of isometric muscle strength, especially immediately after therapy (PostTh5min). Most studies have focused on analyzing these parameters after applying a thermal stimulus. indicating the activity of heat shock protein (HSP) (Van Pelt et al., 2019). HSPs are considered molecular chaperones that play a universal role in maintaining cellular homeostasis (Hoekstra et al., 2018). HSPs have been confirmed to be expressed in skeletal muscle and their induction varies depending on the histological and even functional characteristics of the muscle (Xie et al., 2015). Heat increases gene expression in muscle cell growth and differentiation (Best et al., 2013).

Physical exercise may manifest itself in a transient decrease in muscle strength. Colantuono et al. (2023) which leads to reduced athletic performance (Wang et al., 2022). The intensity of motor discomfort and muscle pain can be eliminated in sports regeneration in many ways, including one of the cheapest and most accessible, which is alternating immersion in warm and cold water (Cochrane, 2004). In our study. both game-ready and partial water immersion therapy brought immediate improvement in isometric muscle strength, with this effect being obvious immediately after and gradually decreasing within an hour after the methods were used (Alexander et al., 2021). In the scientific literature, the most common observations in muscle strength and power provide evidence of its effectiveness, especially in eliminating the effects of strength loss caused by muscle fatigue (Wang et al., 2022). The results of different authors remained heterogeneous, most often up to 72 h after the exercise protocol (S. D. French et al., 2006). Cumulative data in the literature have shown that CWT significantly reduces muscle strength loss at all follow-up times compared to passive recovery (Tseng et al., 2023). The mechanisms of this phenomenon are not fully explained (Wang et al., 2022). CWT is associated with alternating vasodilation and constriction of peripheral blood vessels or “pumping action,” which increases lactate clearance (Kim et al., 2020a), reduces edema (Vaile et al., 2007) and increases blood flow (D. N. French et al., 2008). It is also hypothesized that CWT may alter muscle perfusion by inducing intracellular and intravascular fluid shifts, which may attenuate the immune response and reduce myocellular damage (Cezar et al., 2016). Additionally, the compression used in game-ready can stimulate the receptors responsible for activating the muscle contraction potential. releasing greater muscle force (Valenzuela et al., 2018). Compression probably reduces the oscillatory properties of muscles and has a positive effect on the sensorimotor system, which may explain the beneficial effect on joint proprioception and the sense of position change reported in the literature (Bieuzen et al., 2013). Additionally. there may be a potential increase in arterial blood flow (Bochmann et al., 2005). Alternatively, compression garments are unlikely to significantly alter metabolic responses, while blood pressure, heart rate and cardiorespiratory parameters remained generally unchanged during their use (Yanaoka et al., 2022).

### Limitations and directions of future research

This study presented some limitations. The main limitation is the sample size, as recruiting a more significant, diverse group of combat sports athletes is challenging due to injuries and competition. Given the small sample size, future studies should include a more extensive and diverse sample with extended follow-up periods. Comparing the effects of contrast therapy on stiffness, muscle tone, and elasticity with other post-exercise muscle recovery methods may also provide a valuable reference point for future projects. Moreover, it would be beneficial to study the effect of contrast therapy on people with different levels of physical preparation and practicing different sports disciplines. Another limitation of the study is the need for more current consensus on the best exercise repetition recovery protocol for athletes when using multiple protocols. In this study only male high level fighters were examined, the only reason for this is that we had an excellent access to this group, and gathering high level MMA female athletes was difficult for us. Considering this, future research should include examining women athletes and non-sporting volunteers. Future studies should expand study groups and post-fatigue intervention protocols to evaluate their effectiveness in reducing fatigue.

## Conclusions and practical implications

In summary, this study suggests a positive effect of both contrast therapy strategies as a stimulus influencing important aspects of muscle biomechanics, pain threshold and muscle strength. The results suggest similar outcomes of CWT and GRT, except for the analysis of muscle tone, in which the impact of GRT was statistically more significant. Considering the observed changes of the muscles’ biomechanical parameters measured with myotonometry we confirm the potential of both CWT and GRT to enhance the recovery process. It should be remembered that water therapy has advantages related to availability and low cost in the case of amateur sports, which may be an advantage in some conditions. Despite challenges related to group size and diversity, our findings suggest opportunities for further research into contrast therapy and improved recovery strategies in sports.

## Data Availability

The raw data supporting the conclusion of this article will be made available by the authors, without undue reservation.
